# Medical expenses and its determinants in female patients with urological disorder

**DOI:** 10.1186/s12962-024-00556-x

**Published:** 2024-05-24

**Authors:** Sewon Park, Seokmin Ji, Hyunseo Lee, Hangseok Choi, Mankyu Choi, Munjae Lee, Mihajlo Jakovljevic

**Affiliations:** 1 Department of Medical Science, Ajou University School of Medicine, Suwon, 16499 South Korea; 2 Department of Health Policy & Management, College of Health Science, Korea University, Seoul, South Korea; 3 BK21 FOUR R&E Center for Learning Health Systems, Korea University, Seoul, South Korea; 4 Department of Biostatistics, Korea University College of Medicine, Seoul, South Korea; 5 Medical Science Research Center, Korea University College of Medicine, 73, Goryeodae-ro, Seongbuk-gu, Seoul, South Korea; 6 UNESCO - The World Academy of Sciences (TWAS), Trieste, Italy; 7 Shaanxi University of Technology, Hanzhong, Shaanxi 723099 People’s Republic of China; 8 Department of Global Health Economics and Policy, University of Kragujevac, Kragujevac, Serbia

**Keywords:** Urological disorders, Women’s health, Medical expenses, Chronic conditions care, Prevention, Digital health

## Abstract

**Background:**

The rising older adult population has led to an increase in the prevalence of chronic diseases and medical expenses. Women tend to have a longer healthy life expectancy than men and are more likely to be exposed to urological disorders around the age of 50, resulting in substantial healthcare expenses throughout their lifetime. Urological disorders often require continuous treatment owing to their high risk of recurrence, contributing to an increased financial burden from medical costs. This study aimed to identify factors influencing medical expense in female patients with urological disorders and propose strategies to alleviate the associated financial burden.

**Methods:**

We used data from the Korea Health Panel Survey conducted from 2011 to 2016. The final sample comprised 2,932 patients who visited hospitals for urological disorders. To identify the factors influencing medical expense among female patients with urological disorders, we employed a generalized estimating equation model.

**Results:**

The results indicated that younger people and patients with middle-income levels tended to incur higher medical expenses. Furthermore, patients receiving treatment at tertiary hospitals and those enrolled in National Health Insurance also incurred higher health expenses.

**Conclusions:**

This study suggests that effective management of medical expenses related to urological disorders in women requires improvements in healthcare accessibility to facilitate early detection and continuous disease management. In addition, the findings highlight the potential benefits of digital health and non-face-to-face treatments in addressing these needs.

## Background

Global changes in the population structure due to aging have led to a rise in medical expenses, thereby, increasing the societal burden [1]. In particular, Korea is becoming a super-aged society where the burden of medical expenses caused by aging and low birth rates is soaring [2, 3]. In addition, the number of patients with chronic diseases requiring continuous treatment and management increases with age, with one in three adults suffering from complex chronic diseases, thus requiring systematic management [4, 5]. Meanwhile, the distribution of medical expenses varies depending on age, gradually increasing as one reaches adulthood and, then, increasing rapidly before death [6]. In particular, chronic diseases are a key factor in medical expenses. As the older adult population increases, the prevalence of chronic diseases also increases, and the resulting heightened medical utilization leads to a rise in medical expenses [7–9]. Medical expenses soar for end-of-life patients; if the burden of medical expenses accelerates due to high medical demand, it seems to act as a factor that can hinder the sustainability of health insurance finances, which are responsible for national medical security [10, 11].

The lifespan of women is approximately 3.4 years longer than that of men; accordingly, women spend approximately 19% more on lifetime medical costs than men [12–14]. Furthermore, chronic diseases occur more often in women, and the ensuing medical expense burden appears to be greater in women than in men [13, 15, 16]. Around the age of 50, women are more likely to be exposed to not only chronic diseases but also urological disorders, such as urethritis, frequent urination, urinary incontinence, and so on, caused by menopause, resulting in huge lifetime medical costs [17, 18]. Urological disorders occur more frequently in women (50–60%) than in men. In middle-aged and older adult women, the prevalence decreases, increasing again from the age of 65 years or older. Urological disorders recur in 10% of the cases after menopause; thus, it is important to manage such disorders in women [19–21].

Due to the high rate of recurrence of urological disorders, many patients require continuous treatment through ambulatory care and hospitalization, which could result in additional medical expenses. In the United States, medical expenses for urological disorders amount to $86 billion, and in Europe, the societal cost of such disorders is approaching 58 million euros. In particular, most patients with urological disorders are old and at high risk of developing chronic diseases, which will lower their quality of life if medical expenses rise due to the onset of such disorders. The increase in urological disorders in women as they age aggravates the socioeconomic disease burden on individuals; nevertheless, research on managing the medical expenses of female urological disorders is limited [20, 22–24]. Therefore, it is necessary to not only manage the burden of medical expenses on individual women, measures are needed to reduce such expenses through disease management to address future increases in national medical expenses. In this study, we examine the factors that affect medical expenses in women with urological disorders to seek solutions to reduce such expenses. Thus, we suggest effective management measures for urological disorders that are classified as chronic diseases.

## Materials and methods

### Variables

This study utilized data from the Korea Health Panel for six years from 2011 to 2016. Firstly, to extract women with urological disorders, we identified urological disorders that occur frequently in middle-aged and older adult women. We extracted urological disorders using ICD-10 codes, including patients who received outpatient treatment for urinary stones (N20–N23), cystitis (N30), urinary incontinence (N39.4), and menopausal disorders (N95.1), totaling 4,645 patients. After excluding 1,713 people who did not respond to the questions on their income or household medical expenses, 2,932 people were selected as participants, that is, women with urological disorders.

In the analysis, demographic characteristics, health-related features, and variables related to medical utilization were utilized. For the analysis of factors influencing medical expenditure, commonly used factors such as age, educational level, marital status, income, health insurance, economic activity, disability, and medical institution [9, 24, 25]. Age was divided based on 65 years, and educational level was classified as middle school, high school, and college graduates or higher For marital status, those who were married and living with a spouse were classified as married, and those who were not married or were married but lived alone due to divorce, bereavement, and so on were classified as unmarried. Household income was categorized into quintiles ranging from the 1st (lowest) to the 5th (highest), and health insurance was divided into health insurance subscribers and medical insurance beneficiaries. Those who were currently engaged in economic activities were classified as economic actors and those who were not as non-economic actors. In the case of disability, patients were divided based on whether they had received a disability determination. Medical institutions currently providing ambulatory care were classified as primary care centers, secondary hospitals, and tertiary hospitals. For medical expenses, the dependent variable, that is, the sum up of the total out-of-pocket payments and expenditures on prescription drugs incurred by patients during the treatment period, was applied, and then we applied a logarithmic transformation.

### Statistical analysis

Frequency analysis was conducted to understand the general characteristics of women with urological disorders, and the average medical expenses by group were compared using an independent sample t-test and one-way analysis of variance (ANOVA). In addition, we attempted to identify factors affecting medical expenses for women with urological disorders using a generalized estimating equation model(GEE). GEE is utilized in time-series data, such as panel data, where the same subjects are measured across multiple time periods. By extending the independent generalized linear model to account for repeated measurements of variables, causal relationships between these variables can be estimated. GEE ensures the availability of variability in variables that cannot be observed through cross-sectional regression analysis, allowing for the estimation of various forms of variables. It is deemed suitable for identifying factors influencing medical expenses among women with urological disorders.

## Results

### Demographic characteristics of the patients

The general characteristics of the participants are shown in Table 1. In terms of age, 1,541 (52.6%) and 1,391 (47.4%) people were under and over the age of 65 years, respectively, indicating that the former group had slightly more participants. Regarding education level, 1,464 (49.9%) were middle school graduates or lower, 966 (32.9%) were high school graduates, and 502 (17.1%) were college graduates or higher. Regarding marital status, 821 people (28.0%) were unmarried, whereas 2,111 (72.0%) were married, indicating that most of the participants lived with their spouses. In terms of income, the 1st quintile representing the lowest income accounted for 501 (17.1%), the 2nd quintile for 571 (19.5%), the 3rd quintile for 846 (28.8%), the 4th quintile for 501 (17.1%), and the 5th quintile for 513 (17.5%) people. In terms of economic activity, 1,565 people (53.4%) were not engaged in economic activities, while 1,367 (46.6%) were engaged in economic activities. Regarding the type of medical coverage, 177 (6.0%) people were medical insurance subscribers, and 2,755 (94.0%) had health insurance. In terms of disability, 2,712 participants (92.5%) did not have any disability, whereas 220 (7.5%) did. Regarding the type of medical institution, 2,082 people (71.0%) visited primary care centers, 285 (9.7%) utilized secondary hospitals, and 565 (19.3%) made use of tertiary hospitals.

**Table 1:** General characteristics of the patients

Variables		*N*	%
Age	Under 65	1541	52.6
	65 and over	1391	47.4
Education level	< Middle school graduate	1464	49.9
	≥High school graduate	966	32.9
	≥College graduate	502	17.2
Marital status	Single	821	28.0
	Married	2111	72.0
Household income	1st quintile	501	17.1
	2nd quintile	571	19.5
	3rd quintile	846	28.8
	4th quintile	501	17.1
	5th quintile	513	17.5
Economic activity	No	1565	53.4
	Yes	1367	46.6
Health coverage	Medical aid	177	6.0
	NHI	2755	94.0
Disability	Absent	2712	92.5
	Present	220	7.5
Medical institution	Primary care centers	2082	71.0
	Secondary hospital	285	9.7
	Tertiary hospital	565	19.3

### Comparative analysis of medical expense

An independent sample t-test and one-way ANOVA were conducted to determine whether differences existed in medical expenses based on age, education level, marital status, income, economic activity, type of medical insurance, disability, and type of medical institution (Table 2).

Regarding age, a statistically significant difference was observed in medical expenses (t = 5.002, *p* < 0.001), where those under 65 years had significantly higher expenses than those over 65 years of age. Regarding education level, statistically significant differences were observed in medical expenses (t = 23.782, *p* < 0.001), which appeared to increase in the order of middle school, college, and high school graduates. For marital status, a difference was observed in medical expenses between unmarried and married people (t=-7.581, *p* < 0.001), suggesting that married people had higher medical expenses than unmarried people. In terms of income, differences were observed in medical expenses between the 1st quintile with the lowest income and the 5th quintile with the highest income (t = 32.776, *p* < 0.001). The average medical expense of the income quintile groups was highest in the order of 1st (3.69), 2nd (3.94), 5th (4.00), 4th (4.03), and 3rd (4.04) quintiles. Medical expenses varied depending on participation in economic activities (t=-2.042, *p* = 0.041), in which the group engaged in economic activities had slightly higher expenses than the group unengaged in them. For medical coverage, differences appeared in medical expenses between the group covered by medical insurance and the group covered by health insurance (t=-23.287, *p* < 0.001), where the group covered by health insurance had significantly higher expenses than the one covered by medical insurance. A difference was observed in the medical expenses depending on whether they had a disability (t = 4.015, *p* < 0.001); the group without any disabilities had statistically significantly higher medical expenses than the group with disabilities. Regarding medical institutions, the difference in medical expenses among primary care centers, secondary hospitals, and tertiary hospitals was statistically significant at 0.001 (t = 207.961, *p* < 0.001); the average between groups was in the order of primary care centers (3.82) and secondary (4.16) and tertiary (4.32) hospitals.

**Table 2:** Differences in medical expense based on patient general characteristics

Variables		Medical Expenses (KRW)		
		Mean (SD)	t/F	*p*
Age	Under 65	4.00(0.51)	5.002^***^	< 0.001
	65 and over	3.90(0.62)		
Education level	< Middle school graduate	3.89(0.63)	23.782^***^	< 0.001
	≥High school graduate	4.06(0.50)		
	≥College graduate	3.92(0.47)		
Marital status	Single	3.82(0.67)	-7.581^***^	< 0.001
	Married	4.00(0.52)		
Household income	1st quintile	3.69(0.72)	32.776^***^	< 0.001
	2nd quintile	3.94(0.57)		
	3rd quintile	4.04(0.53)		
	4th quintile	4.03(0.52)		
	5th quintile	4.00(0.46)		
Economic activity	No	3.93(0.63)	-2.042^*^	0.041
	Yes	3.98(0.49)		
Health coverage	Medical aid	3.03(0.85)	-23.287^***^	< 0.001
	NHI	4.01(0.49)		
Disability	Absent	3.97(0.56)	4.015^***^	< 0.001
	Present	3.80(0.66)		
Medical institution	Primary care centers	3.82(0.49)	207.961^***^	< 0.001
	Secondary hospital	4.16(0.50)		
	Tertiary hospital	4.32(0.68)		

*p < 0.05, ***p < 0.001

### Factors influencing medical expense

The factors affecting changes in medical expenses for women with urological disorders include age, educational level, household income, health insurance, and visiting medical institutions (Table 3). Patients over 65 years of age were found to have medical expenses 0.945 times lower than those under 65 years of age. In addition, patients with a college degree or higher and a high school degree had 0.934 times lower and 1.054 times higher, respectively, medical expenses than those with a middle school degree or lower. The medical expenditures of the households belonging to the 4th quintile of household income appeared to be 1.113 times higher than those with low income. The medical expenses of the patients covered by health insurance were found to be 2.347 times higher than those covered by medical care. In addition, the medical expenses for the patients treated at secondary and tertiary hospitals were 1.364 and 1.809 times higher, respectively, than those in primary care centers.

**Table 3:** Results of generalized estimating equation analysis of medical expense for women with urological disorder

Variables	Medical Expenses (KRW)		
	Exp(B)	SE	*p*-value
**Age (ref: Under 65)**			
Over 65	0.945^*^	0.032	0.075
**Education level (ref: Middle school or below)**			
High school or below	1.054^*^	0.032	0.099
College or above	0.934^*^	0.037	0.063
**Marital status (ref: Single)**			
Married	1.043	0.029	0.139
**Household income (ref: 1st quintile)**			
2nd quintile	1.085^**^	0.032	0.010
3rd quintile	1.111^**^	0.036	0.003
4th quintile	1.113^**^	0.037	0.004
5th quintile	1.065^*^	0.036	0.080
**Economic activity (ref: No)**			
Yes	0.994	0.021	0.775
**Health coverage (ref: Medical aid)**			
NHI	2.347^***^	0.095	< 0.001
**Disability (ref: Absent)**			
Present	0.964	0.049	0.450
**Medical institution (ref: Primary care centers)**			
Secondary hospital	1.364^***^	0.044	< 0.001
Tertiary hospital	1.809^***^	0.031	< 0.001

*p < 0.1, **p < 0.05, ***p < 0.001

## Discussion

When examining previous studies analyzing medical expenses among women with urological disorders, Lee, Y. S., & Khan, A. A. (2023) investigated the cost-effectiveness of non-surgical treatment methods for women with urological disorders [26]. Moon, Rena C., et al. (2022) conducted a comparative analysis of medical expenses based on antibiotic prescription patterns among women with urological disorders [27]. These studies solely focused on medical expenses associated with treatment methods, thereby presenting limitations in identifying specific factors that actually influence medical costs. In this study, we aimed to integrate and analyze the factors contributing to medical expenses among women with urological disorders individually, aiming to identify and propose measures to manage potential economic burdens, such as out-of-pocket expenses, and to provide preventive strategies for managing them.

This study’s findings demonstrated that age, household income, type of health insurance, and type of medical institution influenced the medical expenses for women with urological disorders. Medical expenses increased as the age of women with urological disorders decreased. This result seems to be different from those of previous studies that suggest that medical expenses increase with age [28]. In particular, women experience menopause around the age of 50, causing physical changes, urinary tract symptoms, and so on, when they also undergo genitourinary disorders such as cystitis. These urological disorders recur within 6–12 months; therefore, continuous management is required. Owing to the characteristics of the diseases, women have higher medical expenses in their middle age and beyond (under 65 years of age) than in old age [29, 30].

The older the women, the more likely they are to have chronic diseases such as cardiovascular disease, cancer, and diabetes [31]. Women over the age of 65 spend an average of 1.5 times more on medical expenses than those under the age of 65; an increase in medical expenses will be induced by their hospitalization and continuous intake of medication. Owing to increased life expectancy, 90% of older adults will have chronic diseases [32]. Women who develop urological disorders in their middle and old ages are more likely to develop complex chronic diseases as they experience other chronic diseases while growing older, which will incur continuous medical expenses. In other words, as they get older, they come to utilize more medical services, not only for urological disorders but also for other chronic diseases [33–35]. Although they receive positive treatments for their urological disorders in the early stages of the disease, medical expenses for relatively non-life-threatening urological disorders decrease due to the increase in other chronic diseases from aging.

Patients in the middle-income bracket tended to incur higher medical expenses [36]. This finding is similar to previous research results, suggesting that the middle class pays more medical expenses compared to its income difference and that patients with mid-range total household income feel a greater financial burden due to medical expenses [7, 33, 37, 38]. For women, the higher their household expenditure, the higher the prevalence of complex chronic diseases due to their early detection. As medical expenses must be continuously paid to manage the disease, the higher the socioeconomic level, the higher the medical expenses [39]. Even if they show similar disease conditions, those who belong to the middle-income bracket spend 2–3 times more on medical expenses for healthcare than those who belong to the low-income bracket, and when household per capita expenditure increases, medical expenses also increase [40]. This is possibly because the middle-income bracket uses more non-payment medical services that are not covered by health insurance [41].

In addition, these results show a similar context to that of this study: female patients with urological disorders who received treatment at tertiary hospitals will spend more on medical expenses. This finding is supported by previous research, suggesting that patients with chronic diseases, including urological disorders, can receive professional treatment at medical institutions such as tertiary hospitals and must pay the increased medical expenses of receiving more treatments at tertiary hospitals where hospitalization is possible [42]. This is because tertiary hospitals have relatively more non-payment items, and, to proactively deal with a disease in its early stage, they tend to use non-payment items, such as ultrasound, procedures, and so on, for its diagnosis and treatment [43].

Patients covered by health insurance incur higher medical expenses. This finding is similar to the results of previous studies suggesting that health insurance subscribers have higher occurrence factors for spending on medical expenses and that households covered by health insurance have a higher probability of overburdened medical expenses than medical beneficiaries [44, 45]. This is induced by unmet medical care by the type of medical security; 10.03% of health insurance subscribers and 21.59% of medical beneficiaries have experienced unmet medical care. This suggests that patients covered by health insurance are less likely to experience unmet medical care [46]. Furthermore, it may be the result of the difference in that 36.73% of health insurance subscribers are unable to use medical institutions due to lack of time, whereas 54.74% of medical beneficiaries are unable to use medical institutions owing to their economic reasons [25].

### Solutions for reducing medical expenses

Examining the changes in medical expenses according to age, medical expenses are high immediately after birth. The expenditure level gradually decreases until adulthood, continues to increase from the age of 20 to late middle age, and rapidly increases in the older adult population [47, 48]. Considering this, the level of annual per capita medical expenses increases with age and, then, increases relatively faster for advanced age [49, 50]. However, women begin to experience urological disorders from the age of 40 and above, and the prevalence appears to range widely from 5 to 70% in those aged 40 ∼ 60 [51, 52]. As such, urological disorders in women are detected at a relatively young age, but the number of chronic diseases may increase as they get older; therefore, comprehensive disease management is required.

Women with urological disorders are at a higher risk of having comorbidities due to decreased physical activity resulting from involuntary urine leakage, among other factors. Most often, they undergo treatment with medications or non-surgical procedures, and the medical expenses they bear include diagnostic, therapeutic, and ancillary costs for daily management, as well as indirect costs associated with comorbidities. Particularly, women with urological disorders show a continuous increasing trend in low- and middle-income countries, and aging in women leads to a higher incidence of urological disorders compared to men. With life expectancy increasing, the continuous visits to medical institutions for urological disorder management and prolonged stays in long-term care facilities will result in a sustained increase in medical expenses due to medication usage, non-surgical procedures, and other factors. In particular, in women with urological disorders, the recurrence rate is high, which can result in an increased burden of medical expenses on patients.

In this study, when observing that patients covered by health insurance incur higher medical expenses, it can be interpreted that women with urological disorders bear a higher out-of-pocket burden. Thus, it is necessary to prevent medical service accessibility from being hindered by the burden of medical expenses by strengthening coverage and reducing non-payment items for female urological disorders. If the burden of medical expenses is alleviated by increasing the level of health insurance benefits, the burden of medical expenses could be alleviated through the prevention and continuous management of chronic diseases [53]. The dual burden of diseases due to population aging is acting as a cause of increased medical expenses in advanced countries and BRICS as well. In particular, in BRICS, the low public medical expenses suggest a need for strengthening the country’s guarantee, as individual out-of-pocket expenses are high [54]. These results could serve as a basis for strengthening healthcare coverage to enhance healthcare accessibility and promote equity in healthcare provision in countries such as BRICS.

Particularly in the case of women with urological disorders, it is important to detect such disorders at an early stage because the longer the symptoms, the longer the treatment period, which will increase medical expenses. Income and the type of health insurance are closely related, and it is necessary to improve medical service accessibility for the early detection and management of diseases [55]. Accordingly, to reduce long-term medical expenses through disease prevention, women with urological disorders must be managed using digital health and therapeutics. Many cases of women with urological disorders exist where early detection is often difficult owing to the patient’s reluctance to visit medical institutions because of the inconvenience of diagnosis, time, and cost burden. However, urological disorders in women can be prevented by continuously monitoring their health status using digital health and digital therapeutics.

In addition, to manage urological disorders in women, in which the disease duration is inevitably prolonged, it is necessary to expand the treatment for these disorders from a long-term perspective. In the case of chronic diseases such as urological disorders, which are relatively less life-threatening and require continuous management, the utilization of untact treatment is increasing [56]. Because mild urological disorders can be managed with medication, untact treatment through digital health and Information & Communications Technology(ICT) medical devices can improve patients’ accessibility to medical care. Ultimately, this will reduce medical expenses and enable efficient use of medical care [57, 58].

In particular, as women age, the number of chronic diseases, including urological disorders, increases. Therefore, it is essential to consider creating digital health and digital therapeutics covered by health insurance for disease management. Subsequently, if untact treatment is expanded and linked to community care for chronic disease management in older adults, it will ultimately reduce national medical expenses by forming a preventive management system for individuals and lowering unnecessary medical expenses.

### Limitations of study

This study has a limitation in that it did not examine factors influencing medical expenses according to clinical results. It is necessary to examine the prevalence of female urinary diseases and changes in medical expenses according to the results of health examinations or clinical tests such as body mass index (BMI), fertility, urine analysis, ultrasound, or urodynamic study. In addition, the comorbidities of the participants were not included. Future research should identify medical expenses according to comorbidity type for women with urological disorders to ensure continuity of health care. Nevertheless, this study has great significance in that it used the demographic and medical utilization characteristics of women with urological disorders to identify factors in medical expenses and suggested the necessity for national support to prevent and manage such disorders. We hope that the results of this study will be used to establish policies to reduce the burden of medical expenses for women with urological disorders.

## Conclusion

With increased life expectancy, women will live one-third of their lives after menopause. During this period, women’s health deteriorates due to the increased urological disorders; thus, their health management must be continuous. In particular, the number of patients under the age of 65 suffering from one or more chronic diseases has been increasing recently, which suggests that the preventive management of possible chronic diseases as well as urological disorders is necessary for women’s health management. As for women, if the prevalence of urological disorders increases in their middle and old ages and is, thereby, combined with extended life expectancy, the period of living in poor health increases. Considering that women usually live longer than men and spend more on lifetime medical expenses, prevention and early detection of diseases could ultimately alleviate medical expenses. When particularly observing that medical expenses among middle-aged women with urological disorders are higher, it indicates the necessity for early screening and lifestyle improvements utilizing digital health, emphasizing the need for improved treatment methods and effective preventive measures.

## Data Availability

The data used in this study is available to individual researchers or institutions upon approval by the Korea Health Panel Survey (KHPS, https://www.khp.re.kr).
