# Correction to: Medical expenses and its determinants in female patients with urological disorder: Sewon Park

**DOI:** 10.1186/s12962-024-00561-0

**Published:** 2024-06-19

**Authors:** Sewon Park, Seokmin Ji, Hyunseo Lee, Hangseok Choi, Mankyu Choi, Munjae Lee, Mihajlo Jakovljevic

**Affiliations:** 1 Department of Medical Science, Ajou University School of Medicine, Suwon, 16499 South Korea; 2 Department of Health Policy & Management, College of Health Science, Korea University, Seoul, South Korea; 3 BK21 FOUR R&E Center for Learning Health Systems, Korea University, Hyunseo Lee, Seoul, South Korea; 4 Department of Biostatistics, Korea University College of Medicine, Seoul, South Korea; 5 Medical Science Research Center, Korea University College of Medicine, 73, Goryeodae-ro, Seongbuk-gu, Seoul, South Korea; 6 UNESCO - The World Academy of Sciences (TWAS), Trieste, Italy; 7 Shaanxi University of Technology, Hanzhong, Shaanxi 723099 People’s Republic of China; 8 Department of Global Health Economics and Policy, University of Kragujevac, Kragujevac, Serbia

**Correction to: Cost Effectiveness and Resource Allocation (2024) 22:45**

10.1186/s12962-024-00556-x

Following publication of the original article, it came to the attention of the authors that the given and family names of the second author had been erroneously swapped. Namely, the second author name had published as ‘Ji Seokmin’ rather than ‘Seokmin Ji’. The name has since been corrected in the published article. The authors thank you for reading this erratum and apologize for any inconvenience caused.

